# Attenuation of Anxiety-Like Behavior by *Helichrysum stoechas* (L.) Moench Methanolic Extract through Up-Regulation of ERK Signaling Pathways in Noradrenergic Neurons

**DOI:** 10.3390/ph13120472

**Published:** 2020-12-17

**Authors:** Vittoria Borgonetti, Francisco Les, Víctor López, Nicoletta Galeotti

**Affiliations:** 1 Department of Neuroscience, Psychology, Drug Research and Child Health (NEUROFARBA), Section of Pharmacology, University of Florence, Viale G. Pieraccini 6, 50139 Florence, Italy; vittoria.borgonetti@unifi.it; 2 Department of Pharmacy, Faculty of Health Sciences, Universidad San Jorge, 50830 Zaragoza, Spain; fles@usj.es; 3 Instituto Agroalimentario de Aragón-IA2, CITA-Universidad de Zaragoza, 50013 Zaragoza, Spain

**Keywords:** *Helichrysum stoechas*, medicinal plants, herbal medicine, anxiety, BDNF, hippocampus, noradrenergic neurons

## Abstract

The long-term use of anxiolytic and antidepressant drugs can cause a plethora of side effects and the use of complementary and alternative medicine, which is generally considered safer than conventional medicine, is consistently increasing. *Helichrysum stoechas* (L.) Moench methanolic extract (HSE) has shown MAO-A inhibitory properties in previous studies. With the aim of obtaining innovative and safer therapies for mood disorders, this study investigated the potential activity of HSE in the management of anxiety- and depression-related symptoms. HSE showed dose-dependent (30–100 mg/kg p.o.) anxiolytic-like activity in the light dark box and marble burying tests, without any antidepressant-like activity, as shown by the results of the tail suspension test. Additionally, HSE did not have any effect on the modulation of pain, which highlights its selectivity in the control of anxiety-related behavior. At active doses, HSE did not produce any sedative effect or result in impaired motor coordination and memory functions. Western blotting experiments showed the ability of HSE to counteract the reduction in the phosphorylation of ERK44/42, to restore brain-derived neurotrophic factor (BDNF) expression and to return cyclic AMP response element binding (CREB) levels to basal levels in noradrenergic hippocampal neurons of mice exposed to an anxiety-related environment, which indicates a protective role against anxiety behavior. These results suggest that oral administration of HSE might represent an interesting opportunity for the management of anxiety disorders.

## 1. Introduction

Mental disorders are a major contributor to the global burden of disease [1] and depression and anxiety disorders are the most prevalent mental disorders worldwide [2]. The lifetime risk of depression in the overall population is 15–18% [3] with a comparable incidence in high-income countries and low-income and middle-income countries [4]. In 2008, the World Health Organization (WHO) ranked major depression as the third cause of burden of disease worldwide and projected that the disease will rank first by 2030 [5]. There is a high comorbidity between anxiety and depressive disorders, which renders treatment more complex.

The chronic administration of conventionally used antidepressant drugs is characterized by several side effects and the development of tolerance, which limits their prolonged uses [6]. In the last few decades, the use of herbal medicine in the management of mood disorders has become more accepted and there has been a significant rise in the use of natural remedies to treat depression and anxiety. The European Medicines Agency (EMA) and WHO report several plant extracts for the management of anxiety, which possess different mechanism of action, such as *Lavandula angustifolia* Mill. *Crocus sativus* L., *Passiflora incarnata* L. and *Valeriana officinalis* L. [7,8]. Indeed, these products are perceived as safe alternatives to pharmacotherapy, with a lower risk of adverse effects or withdrawal [9,10]. *Helichrysum stoechas* (L.) Moench, commonly known as everlasting flower, belongs to the family of *Compositae*, and grows in the Iberian Peninsula [11]. Traditionally, it has been used for treating influenza or digestive problems [11], but several studies have reported the antimicrobial, anti-inflammatory and antioxidant activities of *Helichrysum* spp. [12,13]. The analysis of the methanolic extract led to the isolation of several components, in particular arzanol, a characteristic and novel pholoroglucinol α -pyrone isolated from *Helichrysum* spp., which possesses a variety of pharmacological activities including acting as an eicosanoid inhibitor on PGE_2_ synthesis and the NF-κB pathway. Moreover, it has other important constituents including caffeic and quercetin derivates, which possess strong antioxidant and anti-inflammatory activities and they have been reported to have beneficial effects on mood disorders [14,15,16].

A recent study showed the inhibitory activity of *Helichrysum stoechas* (L.) Moench methanolic extract on monoamine oxidase A (MAO-A), which is a well-known enzyme with an important role in maintaining the normal function of several modulators of the central nervous system (CNS) that are involved in behavior, pain and cognitive functions [17]. MAO-A inhibitors have been generally used for the treatment of mood disorders, in particular as antidepressant agents [18] and several studies have reported that the effect of MAO-A inhibitors in increasing ERK activation may be an important reason for their pharmacological activity. One example is moclobemide, which increases the neurite development and prevents cell death in serotoninergic neurons through the up-regulation of the ERK pathway [19].

The aim of this study was to investigate the possible antidepressant/anxiolytic effect of a chemically-defined extract obtained from aerial parts of *Helichrysum stoechas* (L.) Moench on different animal models, and its ability to enhance the possible involvement of the ERK pathway in its mechanism of action in the hippocampus and prefrontal cortex.

## 2. Results

### 2.1. HSE Shows Anxiolytic-Like Activity in Mice

**Figure 1:**
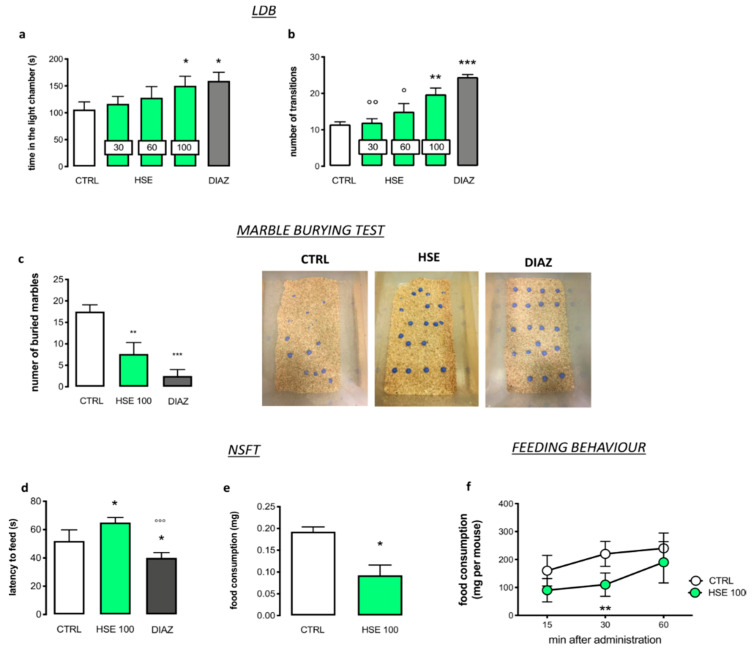
Anxiolytic-like activity of *Helichrysum stoechas* Moench methanolic extract (HSE). (**a**,**b**) In the light dark box (LDB) test, HSE 100 reduced the time spent in the light chamber (**a**; one-way ANOVA post-hoc Tukey, F(4,35) = 14.52, * *p* < 0.05 vs. CTRL) and an increase in the number of transitions (**b**; one-way ANOVA post-hoc Tukey, F(4,35) = 15.55, *** *p* < 0.001 ** *p* < 0.01 vs. CTRL; °° *p* < 0.01, ° *p* < 0.05 vs. DIAZ) compared to mice with anxiety-like symptoms (CTRL) and diazepam (DIAZ). (**c**) HSE 100 reduced the number of buried marbles compared to CTRL (one-way ANOVA post-hoc Tukey, F (2,21) = 116.4, *** *p* < 0.001 ** *p* < 0.01 vs. CTRL). (**d**,**e**) In the novelty suppressed feeding test (NFST) HSE 100 increased the latency to feed (**d**; one-way ANOVA post-hoc Tukey F(2,21) = 42.36, * *p* < 0.05 vs. CTRL, °°° *p* < 0.001 vs. DIAZ) and reduced food consumption (**e**; Student’s *t*-test, * *p* < 0.05 vs. CTRL). (**f**) The food consumption was evaluated with the feeding behavior (two-way ANOVA post hoc Sidak, F(2,24) = 7.176, ** *p* < 0.01 vs. CTRL). The vertical bars represent ± SD of eight animals per group (*n* = 8).

The effect of *Helichrysum stoechas* Moench methanolic extract (HSE) on anxiety-related behavior was primarily investigated by using the light-dark box (LDB) test. Dose-response experiments showed that mice treated with HSE at a dose of 100 mg kg^−1^ spent significantly more time in the light chamber compared to the control group (CTRL), thus highlighting its anxiolytic-like properties. The effect was of similar intensity to that produced by diazepam (DIAZ), which was used as a reference drug. The dose of 30 mg kg^−1^ showed values comparable to the control group. While not statistically significant (*p* = 0.4716), an increase in the time in the light chamber was observed with 60 mg kg^−1^ (HSE 30: 116 ± 13.72 s; HSE60 128.1 ± 20.63 s; HSE 100: 150.3 ± 17.62 s; DIAZ: 160 ± 15.92 s vs. CTRL 106.00 ± 14.32 s) (Figure 1a). A similar trend was observed for the number of transitions between the light and dark boxes, which represents another parameter for investigating the anxiolytic-like effect of a substance (Figure 1b). Indeed, HSE 100 mg kg^−1^ increased the number of transitions in comparison to the CTRL group while no statistical difference was observed for HSE 30 and 60 mg kg^−1^ (HSE 30: 12.00 ± 2.93; HSE60 15.00 ± 6.27; HSE 100 19.71 ± 4.84; DIAZ: 24.50 ± 1.85 vs. CTRL 11.50 ± 1.97). As a consequence, HSE was used at a dose of 100 mg kg^−1^ for all of the other experiments. In the marble burying test, the higher the number of marbles buried, the higher the state of anxiety of mice [20]. As reported in Figure 1c,d, the number of marbles buried by the animals treated with HSE was significantly lower than the CTRL, and the anxiolytic activity was similar to DIAZ (HSE 100: 7.87 ± 2.66; DIAZ: 2.57 ± 1.51 vs. CTRL: 17.50 ± 1.60).

The novelty suppressed feeding test (NSFT) was used to better investigate the anxiolytic activity of HSE [21]. The data obtained showed a longer latency to feed in HSE-treated animals compared to the CTRL. Conversely, treatment with DIAZ decreased the feeding latency (HSE 100: 65.00 ± 3.54; DIAZ: 40.00 ± 3.78 vs. CTRL: 52.00 ± 7.85) (Figure 1e), indicating an anxiogenic response for the extract. However, the HSE group showed a reduced amount of food consumption compared to the CTRL and the induction of an anorexiant effect (HSE 100: 0.09 ± 0.05 vs. CTRL: 0.19 ± 0.031) (Figure 1f) might mask the anxiolytic activity of HSE, leading to a negative result in this test. The anorexiant activity was better confirmed with the feeding behavior test, where mice treated with HSE 100 mg kg^−1^ were previously food-deprived for 4 h. Figure 1f represents the curve for the food eaten by CTRL and HSE-treated mice. The CTRL mice show a progressive increase in food intake in 60 min. The HSE group show very low food consumption during the test. The cumulative amount of food eaten was significantly lower than the CTRL, and showed an anorexic effect which peaked coincidently with the peak of anxiolytic activity and then disappeared 60 min after treatment (15 min-HSE 100: 90.00 ± 41.43 s vs. CTRL: 160.00 ± 54.77; 30 min-HSE100: 110.00 ± 41.83 s vs. CTRL: 220.00 ± 44.72 s; 60 min HSE100: 190.00 ± 74.16 s vs. CTRL: 240.00 ± 54.77 s).

### 2.2. Antidepressant-Like Activity of HSE in a Depressant-Like Paradigm

**Figure 2:**
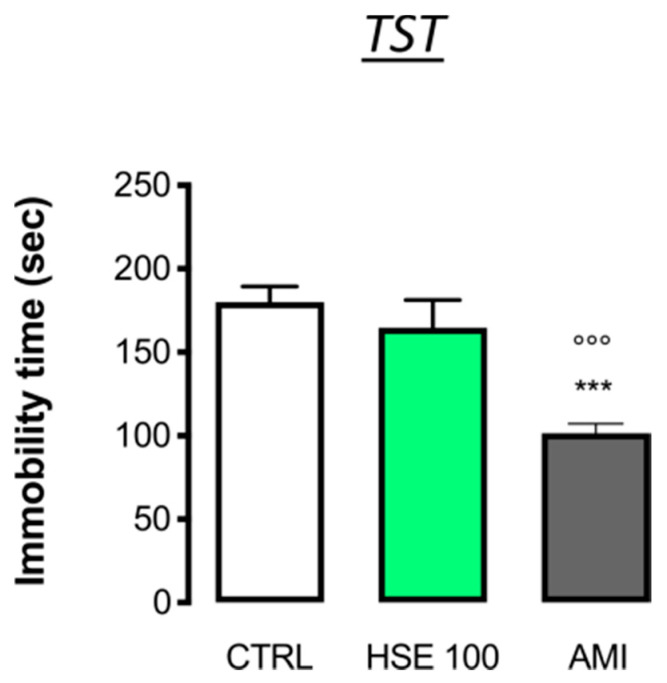
Evaluation of the antidepressant-like activity of *Helichrysum stoechas* Moench methanolic extract (HSE) in the tail suspension test (TST) compared to amitriptyline (AMI) (one-way ANOVA post-hoc Tukey F (2,12) = 64.05, *** *p* < 0.001 vs. CTRL, °°° *p* < 0.001 vs. AMI) The vertical bars represent ± SD (*n* = 8).

To investigate the effect of HSE in a depressant-like behavior test, the tail suspension test (TST), a common test used to verify the anti-depressant like effect of novel drug candidates, was used [22]. HSE did not alter the immobility time compared to the CTRL group during the last 4 min of the test, i.e., when the depression appears, thus, excluding any antidepressant-like activity. Indeed, the administration of amitriptyline (AMI), a reference antidepressant drug, reduced the immobility time values compared to the CTRL (HSE100: 164.80 ± 16.80 s; AMI: 101.6 ± 5.48 s vs. CTRL 180.00 ± 9.52 s) (Figure 2).

### 2.3. Lack of Analgesic Activity by HSE

**Figure 3:**
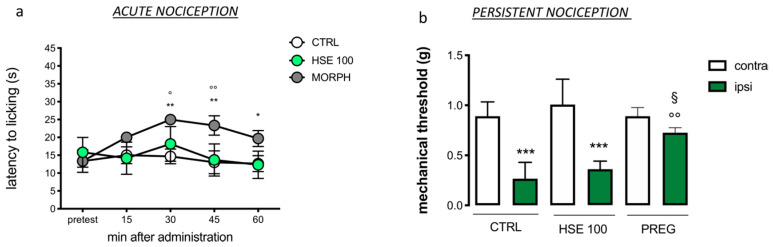
Antinociceptive profile of *Helichrysum stoechas* Moench methanolic extract (HSE) against an (**a**) acute thermal stimulus (hot plate test) in comparison with control group (CTRL) and morphine (MORPH) (**a**, two-way ANOVA post-hoc Sidak, F(4,75) = 6.753, ** *p* < 0.01 * *p* < 0.05 vs. CTRL; °° *p* < 0.01, ° *p* < 0.05 vs. HSE 100) and against (**b**) mechanical stimulus (von Frey hairs) in a spared nerve injury (SNI) model (**b**; two-way ANOVA post-hoc Sidak, F (5,30) = 26.59, *** *p* < 0.001 vs. contra; °° *p* < 0.01 vs. HSE 100 ipsi; § *p* < 0.05 vs. CTRL). The vertical bars represent ± SD (*n* = 6).

The antinociceptive activity of HSE was evaluated in models of acute and persistent pain and the modulation of the pain threshold was measured against a thermal (i.e., the hot plate test) and mechanical (i.e., the von Frey’s test) stimulus. Time-course experiments using HSE 100 mg kg^−1^ did not show a statistically significant modulation of the pain threshold in the acute nociceptive model (Figure 3a). No differences in the latency to licking values were observed, compared to the CTRL. Conversely, morphine (MORPH), used as reference drug, increased the mouse pain threshold (pretest-HSE100: 15.83 ± 4.16; MORPH: 13.33 ± 3.14 vs. CTRL 13.33 ± 3.18; 15 min-HSE100: 14.16 ± 4.49, MORPH 20.00 ± 0.577 vs. CTRL: 15.00 ± 1.53; 30 min-HSE100: 18.16 ± 1.99, MORPH 25.00 ± 0.58 vs. CTRL: 14.67 ± 1.33; 45 min-HSE100: 13.67 ± 4.83, MORPH: 23.33 ± 1.77 vs. CTRL 13.00 ± 3.22; 60 min-HSE100: 12.33 ± 1.57, MORPH: 19.67 ± 2.25 vs. CTRL:12.67 ± 2.25). In order to completely exclude the antinociceptive activity of the extract, HSE was also tested in mice in a model of neuropathic pain, the spared nerve injury (SNI) model, which is characterized by the presence of persistent pain (Figure 3b). This model produces a rapid and long-lasting mechanical allodynia on the ipsilateral side compared to the contralateral uninjured side. Seven days after surgery, when pain hypersensitivity is well established, HSE treatment did not prevent mechanical allodynia in the ipsilateral side, compared to the contralateral side, showing values similar to the CTRL group. In this experiment, a group of animals was treated with pregabalin (PREG), used as reference drug, which completely prevented the allodynia in the ipsilateral side compared to the CTRL group. (CTRL-contra 0.89 ± 0.14, ipsi 0.27 ± 0.16; HSE100-contra 1.00 ± 0.25 HSE100-ipsi 0.36 ± 0.08; PREG-contra 0.89 ± 0.086, PREG-ipsi 0.72 ± 0.05).

### 2.4. Lack of Locomotor Behaviour Impairment

**Figure 4:**
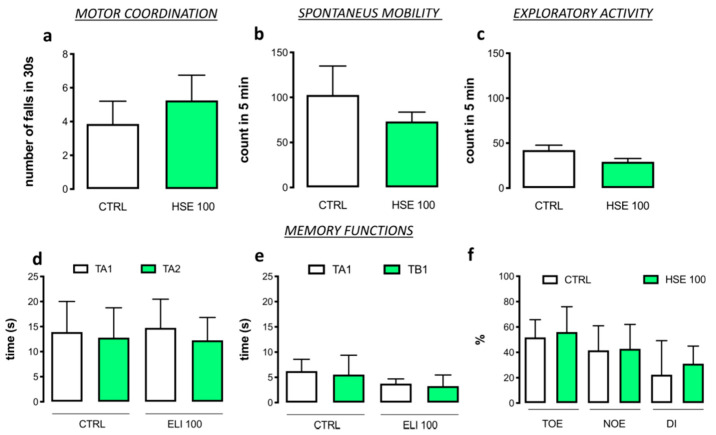
Evaluation of *Helichrysum stoechas* Moench methanolic extract (HSE) effects on (**a**), motor coordination, (**b**) spontaneous mobility, (**c**) exploratory activity and on (**d**–**f**) memory functions compared to the CTRL group (mice with anxiety-like symptoms) (Student’s *t*-test). The vertical bars represent SD (*n* = 8).

Mice treated with HSE 100 mg kg^−1^ did not show any alteration in gross behavior. In addition, specific tests were conducted to investigate possible locomotor alterations that were not visible to the operator. Figure 4a shows the effect of HSE on motor coordination measured with rotarod test. There was no significant increase in the number of falls in 30 s in the HSE group compared to the CTRL group (HSE100: 5.25 ± 0.75 falls vs. CTRL: 3.86 ± 0.51 falls). The hole board test was used for measuring the spontaneous mobility (HSE100: 73.25 ± 5.21 planes vs. CTRL: 102.90 ± 0.12 planes) (Figure 4b) and exploratory activity (HSE100: 29.25 ± 3.68 holes vs. CTRL: 42.29 ± 5.55 holes) (Figure 4c) of treated mice, which were unaltered by HSE administration in comparison with CTRL group.

### 2.5. No Alteration of Memory Function by HSE

The effect of HSE treatment on memory processes was investigated using the novel object recognition test (NORT). No differences were observed between HSE-treated mice and the CTRL group during the training (CTRL: TA1 = 13.90 ± 1.37, TA2 = 12.80 ± 1.33; HSE 100 TA1 = 14.75 ± 2.87, TA2 = 12.25 ± 2.29) (Figure 4d) and the retention session. (CTRL: TA1 = 6.23 ± 0.56 TB1 = 5.55 ± 0.90; HSE 100: TA1 = 3.75 ± 0.47 TB1 = 3.25 ± 1.10) (Figure 4e). Thus, HSE-treated mice did not show any alteration in memory function compared to the CTRL group, as demonstrated by the training object exploration index (TOE), the novel object exploration index (NOE), and the discrimination index (DI) (TOE: CTRL = 51.57 ± 3.14 HSE 100 = 56.00 ± 9.98; NOE: CTRL = 41.65 ± 4.31 HSE 100 = 42.75 ± 9.63; DI: CTRL = 22.40 ± 5.99 HSE 100 = 31.00 ± 6.98) (Figure 4f).

### 2.6. HSE Increased p-ERK44/42 and Brain-Derived Neurotrophic Factor (BDNF) Expression in the Hippocampus

**Figure 5:**
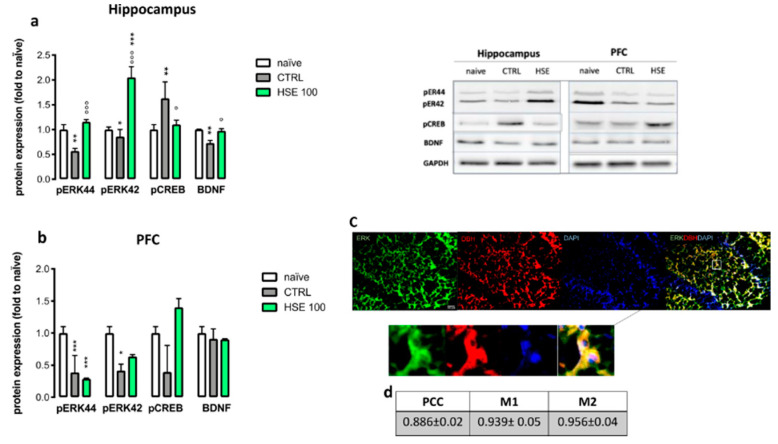
(**a**) *Helichrysum stoechas* Moench methanolic extract (HSE) increased the phosphorylation of ERK44 in the hippocampus (one-way ANOVA post-hoc Tukey, F(2,6 = 57.14) ** *p* < 0.01 vs. naïve; °°° *p* < 0.001 vs. CTRL) and ERK42 (one-way ANOVA post-hoc Tukey, F(2,6 = 56.88), *** *p*< 0.001 * *p* < 0.01 vs. naïve; °°° *p* < 0.001 vs. CTRL) compared to naïve (mice without anxiety-like symptoms) and CTRL (mice with anxiety-like symptoms). HSE reduced the pCREB (one-way ANOVA post-hoc Tukey, F(2,6 = 8.157), ** *p* < 0.01 vs. naïve; ° *p* < 0.05 vs. CTRL) and increased BDNF (one-way ANOVA post-hoc Tukey, F(2,6 = 43.47), ** *p* < 0.01 vs. naïve; ° *p* < 0.05 vs. CTRL) compared to the CTRL group. (**b**) HSE did not show any effects on p-ERK42/44 in the prefrontal cortex (PFC) (one-way ANOVA post-hoc Tukey, F(2,6) = 37.98, *** *p* < 0.001 * *p* < 0.05 vs. naïve), pCREB (one-way ANOVA post-hoc Tukey, F(2,6) = 11.65)) and BDNF (one-way ANOVA post-hoc Tukey, F(2,6) = 11.0.766) protein levels, compared to the CTRL group. Representative micrograph showing ERK (green), DBH (red), DAPI (blue) and merge (**c**). Pearson’s correlation coefficient (PCC) and M1/M2 values are reported in the table (**d**). The vertical bars represent ± SD (*n* = 3). Representative blots are reported.

In the hippocampus of mice with anxiety-like symptoms (i.e., CTRL), a reduction of protein levels of phosphorylated extracellular signal-regulated kinase (p-ERK)44, p-ERK42 and BDNF was detected as soon as there was an increase in cyclic AMP response element binding (CREB) levels compared to naïve mice. HSE treatment completely prevented the anxiety-induced reduction of p-ERK44 and BDNF and the increase of CREB, and brought the levels back to the naïve group ones (Figure 5a). Moreover, a strong up-regulation of the protein levels of p-ERK42 was induced compared to the naïve group. Similarly, in the prefrontal cortex (PFC) a reduction of p-ERK44 and p-ERK42 was observed in the CTRL group. On the contrary, no significant differences were observed for cyclic CREB and BDNF levels, thus, suggesting a selective effect in the hippocampus (Figure 5b). To confirm the involvement of adrenergic neurons in the anxiolytic-like effect of HSE, double labelling immunostaining with dopamine β-hydroxylase (DBH), a classical marker of noradrenergic neurons [23], and p-ERK44/42 was performed. Merged images showed a strong colocalization between p-ERK and DBH (Figure 5c) that was confirmed by high colocalization coefficient values from quantification analysis (Figure 5d).

## 3. Discussion

Nowadays, the use of complementary and alternative medicine in the management of psychiatric disorders is a common phenomenon [10]. The interest in using herbal extracts has been promoted by the concept that these products are generally considered safer than conventional medicine. Indeed, the long-term use of anxiolytic drugs can cause a plethora of side effects together with an increase in drug tolerance, which has a drastic influence on the patients’ quality of life [24]. *Helichrysum stoechas* (L.) Moench belongs to the *Helycrisum* genus, and differently to other species of the same family, its pharmacological potential has not been completely investigated. Indeed, only its antioxidant and antimicrobial properties have been studied [25]. Recently, Les et al. [17] reported the in vitro dose-dependent inhibition of MAO-A enzyme activity, which is an important target in the management of mood disorders [18], using a chemically characterized *Helichrysum stoechas* methanolic extract (HSE). For this reason, the aim of this work was to investigate the potential effect of the above-mentioned extract in the management of anxiety- and depression-related symptoms in animal models.

In our experiments, we demonstrated that the acute administration of HSE is able to increase the time spent in the light chamber compared to the CTRL group. This test is commonly used to assess anxiety-related response and the possible anxiolytic-like effect of a drug candidate. Moreover, HSE also showed anxiolytic activity in the marble burying test with HSE-treated mice showing a trend whereby the number of buried marbles was reduced, which confirms the anxiolytic-like effect observed in the LDB. This effect is similar to that observed with diazepam, which was used as a reference drug [26]. These results are consistent with the work of De Angelis and Furlan [27], where moclobemide, a well-known MAO-A inhibitor [28], showed anxiolytic-like effects in the LDB test. Similarly, Nicolas and colleagues [29] reported the effectiveness of phenelzine (a non-selective MAO inhibitor) in reducing the number of buried marbles compared to the CTRL group.

However, a decrease in the tendency to reduce latency to feed in the NSFT was observed, which is in contrast with data reported for conventional anxiolytic-like drugs. This discrepancy can be explained by considering that a reduction in the amount of eaten pellet in mice treated with HSE was observed at the peak of the effect. This anorexiant effect could explain why mice spent more time getting to the food, compared to the CTRL and diazepam-treated groups. Indeed, the potent anorectic effect of MAO inhibitors in animal models is well documented and it has already been evaluated as a possible therapeutic opportunity to treat obesity [30]. Contrary to common anxiolytic drugs, HSE reduced food consumption at active doses, which represents a clinical advantage for long-term therapies.

Despite being commonly perceived as distinct psychiatric disorders that are believed to be caused by alterations of different brain circuits, there is a high comorbidity between anxiety and depression [31], and often, anxiety disorders are underdiagnosed and undertreated in primary care [32]. Furthermore, commonly used anxiolytic drugs, such as benzodiazepines, in addition to controlling anxiety, are able to induce several additional activities (i.e., sedation, muscle-relaxation, etc.). HSE did not induce sedation or cognitive impairment, which is a clinical advantage for long-term therapies.

HSE did not show any anti-depressant-like effect in the TST, and did not induce sedation or alteration of locomotor behavior, which suggests HSE is a selective anxiolytic agent. Moreover, the lack of locomotor impairment or excessive sedative effects indicates that HSE has a good safety and tolerability profile.

It is also commonly accepted that anxiolytic drugs can have analgesic properties [33,34,35]. Interestingly, HSE showed anxiolytic-like effect that was devoid of any efficacy in the control of pain, further highlighting its selectivity in the control of anxiety-related behavior.

The decrease of BDNF expression in the brain, especially in the hippocampus and PFC is associated with anxiety [36]. Indeed, the effect of some anxiolytic drugs can include the increase of neurotrophins [37]. HSE administration increased BDNF in the hippocampus, but not in the PFC, thus showing a region-specific effect. BDNF is tightly connected with the activation of the ERK signaling pathway [38], and our results highlighted this relationship. A reduction of ERK44/42 phosphorylation, linked to a reduction of BDNF levels, in mice exposed to an anxiety-related environment was observed. HSE reverted this effect, and induced an increase in the phosphorylation of ERK44/42. It has also been reported that the CREB level in the brain is a key factor in modulating anxiety-related behaviors, and its activation is related to increased anxiety-related symptoms [39]. HSE reduced CREB levels in the hippocampus, suggesting a protective role against anxiety behavior. However, an opposite effect on CREB activation has been observed in the prefrontal cortex, where the levels of p-CREB have been increased by the treatment. Several studies have reported that the activation of CREB in different brain regions provoked different effects (doi:10.1016/j.tins.2005.06.005). The activation of this transcription factor in the prefrontal cortex could be independent of the ERK-pathway and involved in other effects that are not related to anxiety (doi:10.3389/fnmol.2018.00255).

Hence, the anxiety-like effect of HSE seems to be related to a local hippocampal increase in ERK signaling, together with a reduction in CREB and the restoration of BDNF levels.

All these events appear to occur in noradrenergic neurons, as suggested by the high degree of colocalization between p-ERK and DBH. The modulation of hippocampal noradrenergic neurons activity is consistent with the promotion of an anxiolytic-like effect. In fact, MAO inhibitors are used as potential treatments for patients with generalized anxiety disorder (GAD) [40].

## 4. Materials and Methods

### 4.1. Animals

**Figure 6:**
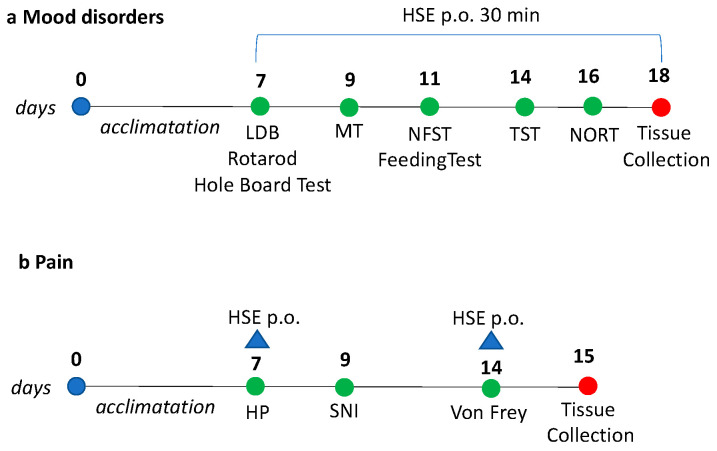
(**a**) Schematic representation of behavior experiments (LDB: light dark box; MT: marbles test; NFST: novelty suppressed feeding test; TST: tail suspension test; NORT: novel object recognition test). (**b**) Schematic representation of pain experiments (HP: hot plate; SNI: spared nerve injury).

CD1 male mice (4–6 weeks of age) weighing approximately 22–24 g (Envigo, Varese, Italy) were kept in the Ce.S.A.L. (Centro Stabulazione Animali da Laboratorio, University of Florence) vivarium and used five days after their arrival. Mice were housed in standard cages, kept at 23 ± 1 °C with a 12-h light/dark cycle, light on at 7 a.m., and fed with standard laboratory diet and tap water ad libitum. The cages were placed in the experimental room 24 h before behavioral testing for acclimatization. All tests were conducted during the light phase. The experimental protocol was approved by the Institution’s Animal Care and Research Ethics Committee (University of Florence, Florence, Italy), under license from the Italian Department of Health (54/2014-B). Mice were treated in accordance with the relevant European Union (Directive 2010/63/EU, the Council of 22 September 2010 on the protection of animals used for scientific purposes) and international regulations (the Guide for the Care and Use of Laboratory Animals, U.S. National Research Council, 2011). All studies involving animals are reported in accordance with the ARRIVE guidelines [41]. The experimental protocol was designed to minimize the number of animals used and their suffering and it has been graphically represented in Figure 6. Power analysis was used to determine the number of animals per experiment [42]. The number of animals per group were decided in order to have a probability of 86%, at which the study detects the difference between groups (0.05 significance level). G power software was used to calculate the sample size.

### 4.2. Plant Material

*Helichrysum stoechas* (L.) Moench methanolic extract (HSE) was obtained from the aerial parts, including flowers, by cold maceration with methanol as previously reported [17,43]. The solvent was removed by using rota-vaporing techniques. The main constituents identified in this extract and in previously published studies were of a phenolic nature: arzanol, α-pyrone, helipyrone, three phenolic acids (p-hydroxybenzoic, caffeic and neochlorogenic acids), and flavonoid derivatives (5,7-dihydroxy-3,6,8-trimethoxyflavone, isoquercitrin and quercetagetin-7-*O*-glucopyranoside) [12].

### 4.3. Chemicals and Drug Administration

HSE was dissolved in 5% DMSO and orally administered (p.o.) 30 min before testing at a dose of 30, 60 and 100 mg/kg. Diazepam (DIAZ, 1 mg/kg i.p.) and amitriptyline (AMI, 10 mg/kg i.p.) (Sigma Aldrich, Milan, Italy) were administered 30 min before testing. Morphine hydrochloride (MORPH 7 mg/kg i.p.) (SALARS, Como, Italy) was administered 15 min before testing and pregabalin (PREG 30 mg/kg i.p.) (Sigma Aldrich) was administered 3 h before testing. The above-mentioned reference drugs were dissolved in saline solution.

### 4.4. Evaluation of Anxiolytics Activity

#### 4.4.1. Light Dark Box (LDB)

The light dark (LDB) box apparatus consisted of a rectangular box measuring 50 × 20.5 × 19 cm, divided into two different compartments, one dark (black) and one illuminated by a 60-W bulb lamp (white). These two different areas were divided with a dark insert including a small door (10 × 3.2 cm) at floor level, allowing animals to move freely from one compartment to another [44]. Each mouse was released in the center of the light compartment with its head facing away from the door. The time spent in the light chamber and the number of full-body transitions between chambers was recorded as a marker of anxiety behavior. Then, animals were removed from the L/D box and returned to their home cage in the colony room. After each test, the apparatus was cleaned with 70% ethanol to remove the olfactory cues and allowed them to dry before the next subject was tested.

#### 4.4.2. Marble Burying Test

The marble burying behavioral test was performed as previously described [45]. Briefly, mice were individually placed in clear cages (27 × 16 × 14 cm), each containing 20 glass marbles (1 cm diameter) equidistantly located in a 5 × 4 arrangement. Before testing, each mouse was allowed to freely walk around the cage for 5 min, without marbles. The number of marbles buried (at least two-thirds) in 30 min was recorded. This parameter represent a measure of animal anxiety. The observer did not know which agent was being tested.

#### 4.4.3. Novelty Suppressed Feeding Test and Evaluation (NSFT) of Food Consumption

The NSFT test was performed as previously described [46]. Animals were acclimated for 10 min to the box and deprived of food overnight (12 h). The day of the test mice was individually placed in a corner of the box, in which at the center was put a single pellet of food. The latency to feed and the quantity of pellet eaten, expressed in mg was recorded in 5 min. The observer did not know which agent was being tested. For the feeding behavior, animals were kept fasted for 4 h, with water ad libitum. The difference between the given weight of standard laboratory pellets and the weight of pellet left after 15, 30, and 60 min from administration was recorded.

### 4.5. Evaluation of Antidepressant Activity

#### The Tail Suspension Test (TST)

The TST was performed as described by Sanna and colleagues (2018) [47]. Briefly, mice were suspended 50 cm above the floor on a plastic rod by applying adhesive tape to the upper middle of the tail. The immobility time was measured during a test period of 6 min. The depressant-like behavior was established in the last 4 min, when mice hung passively and completely motionless.

### 4.6. Evaluation of Antinociceptive Activity

#### 4.6.1. Hot Plate Test (HPT)

The hot plate test was performed following the protocol described by Sanna et al. [48]. Mice were placed on a hot plate (Ugo Basile Biological Research Apparatus, Varese, Italy), with the temperature adjusted to 52.5 ± 0.1 °C. The time to the first sign of nociception (paw licking) was recorded before and after treatment administration and the mouse was removed from the hot plate immediately after. To avoid damage to the paws, an arbitrary cut-off time of 45 s was adopted.

#### 4.6.2. Spared Nerve Injury (SNI) Procedure and Von Frey Test (VFT)

Behavioral testing was performed before surgery to establish a baseline for comparison with postsurgical values. This animal model of mono-neuropathy was performed as previously described [49]. Only the right paw (ipsi) was operated on, while the left remained intact (contra). Seven days after SNI operation, the mechanical allodynia was measured by using a Dynamic Plantar Aesthesiometer (Ugo Basile, Gemonio, Italy) as previously described [49]. The mice were placed in individual Plexiglas chambers [8.5 × 3.4 × 3.4 (h) cm]. After a 1 h familiarization period, the mechanical threshold was recorded by delivering a mechanical stimulus using grade-strength von Frey monofilaments (0.004, 0.07, 0.16, 0.4, 0.6, 1.0, 1.4, 2.0 g) on both the ipsilateral and contralateral sides. Monofilaments were delivered to the plantar surface of the hind paw of the mouse, starting with filament of 0.008 g and a positive response was determined by a paw withdrawal response to any two of 5 repetitive stimuli. In case of negative response, the next filament was applied. The averages of the responses were calculated.

#### 4.6.3. Rotarod Test

The possible onset of motor side effects induced by treatment was evaluated with the rotarod test, as previously described [48]. The number of falls in 30 s was registered and used as an indication of motor function.

#### 4.6.4. Hole Board Test

The hole board test is commonly used to verify the effect of a drug on spontaneous mobility and exploratory activity [50]. The hole board consists of a wooden box with 16 holes on the ground. Each animal was evaluated individually for a period of 5 min. During this time four photo beams crossing the plane from midpoint to midpoint of opposite sides registered the movements of the animal on the plane (spontaneous mobility). Each hole contains miniature photoelectric cells that record the head-dips in the holes by the mice, representing the exploratory activity of mice.

### 4.7. Evaluation of Memory Functions

#### Novel Object Recognition Test (NORT)

Memory function was assessed by using the novel object recognition test (NORT), which evaluates the ability of the animals to recognize a novel object in the environment, thus, measuring a form of recognition memory [51]. NORT was performed as previously described [44]. Briefly, a plexiglass box (78 × 60 × 39 cm) was used where mice were allowed to explore the open field. During the familiarization session, the box was empty, with no objects inside. In the first session, the training phase, animals were exposed to identical objects (A1 and A2), placed 16 cm from the wall and 37 cm apart, for 5 min. Object exploration (i.e., sniffing or touching the object with the mouth or nose) was manually measured by an experienced observer, who was blind to the drug treatment. Animals were placed back in the NORT system after 3 h (short-memory) or 24 h (long-memory) from the training session, with two different objects, the familiar A1 (the same as the training phase) and a novel object B for 5 min (test phase), which were always located in the same position. Velcro was applied to the base of the objects, to avoid unwanted movement of the objects. The two objects A1 and B were different in shape, color, and size. To eliminate the olfactory cues, the cage was cleaned with ethanol after each test. The test phase reflects the preference for the novel object. The recognition index for the novel object was calculated using the following formula:

(TB − TA1/TB + TA1) × 100

where TA1 represent the time spent exploring familiar object and TB is the time spent exploring the novel object.

### 4.8. Western Blotting Analysis

Western blotting was performed as previously reported [52]. The dissected hippocampus and prefrontal cortex (PFC) tissue were homogenized in a lysis buffer containing 25 mM Tris-HCl pH (7.5), 25 mM di NaCl, 5 mM EGTA, 2.5 mM EDTA, 2 mM NaPP, 4 mM PNFF, 1 mM di Na3VO4, 1 mM PMSF, 20 µg/mL leupeptin, 50 µg/mL aprotinin, 0.1% SDS (Sigma-Aldrich, Milan, Italy). The homogenate was centrifuged at 12,000× *g* for 30 min at 4 °C and the pellet was discarded.

Protein samples (40 µg of protein/sample) were separated by 10% SDS-polyacrylamide gel electrophoresis (SDS-PAGE). Proteins were then blotted on to nitrocellulose membranes (120 min at 100 V) using standard procedures. Membranes were blocked in PBST (PBS with 0.1% Tween) containing 5% non-fat dry milk for 120 min and incubated overnight at 4 °C with primary antibodies anti p-ERK 1/2 phosphorylated on Thr202/Tyr204 (1:500), anti CREB (1:500) (Cell Signaling), anti-BDNF (1:1000) (Santa Cruz) and The blots were rinsed three times with PBST and incubated for 2 h at room temperature with HRP-conjugated mouse anti-rabbit (1:3000) (Santa Cruz Biotechnology Inc., Santa Cruz, CA, USA) and goat anti-mouse (bs-0296G, 1:5000) (Bioss Antibodies Inc., Woburn, MA, USA) and then detected by chemiluminescence detection system (Pierce, Italy). Signal intensity (pixels/mm^2^) was quantified using ImageJ (NIH). The signal intensity was normalized to that of GAPDH (1:5000 sc-32233) (Santa Cruz Biotechnology Inc., Santa Cruz, CA, USA).

### 4.9. Immunofluorescence

Animals were perfused transcardially with 4% paraformaldehyde in 0.1 M phosphate buffer (PBS, pH 7.4) on day 7. After perfusion, the hippocampus was quickly removed, postfixed for 24 h with the same fixative at 4 °C, and transferred to a 30% sucrose solution for 48 h [53]. After preincubation in 5 mg/mL BSA/0.3% Triton-X-100/PBS, sections were incubated overnight at 4 °C with the primary antibodies, at optimized working dilution. Primary antibodies used were antibodies for anti p-ERK 1/2 (1:500) (Cell Signaling) and DBH (1:300) (Santa Cruz Biotechnology Inc., Santa Cruz, CA, USA). Then, sections were washed three times with PBS containing 0.01% Triton-X-100 and incubated for 2 h at room temperature in secondary antibodies [Alexa Fluor 488 (490–525, 1:400; Thermo Fisher Scientific, Waltham, MA, USA), Alexa Fluor 568 (578–603, 1:400; Thermo Fisher Scientific)]. Finally, sections were covered with Vectorshield mounting medium (Vector Laboratories, Burlingame, CA, USA). The images were acquired with 10× magnification using a Leica DM6000B fluorescence microscope equipped with a DFC350FX digital camera with appropriate excitation and emission filters for each fluorophore. Colocalization of two different labels was measured using EzColocalization plugin (ImageJ), as previously described [54]. The extent of colocalization was determined by calculating the Pearson’s correlation coefficient (PCC) characterized by a range of value between −1 (anti-colocalization) and 1 (complete colocalization) and Mander’s overlap coefficient (M1, M2), characterized by a range of values between 0 (complete anti-colocalization) and 1 (complete colocalization) [55].

### 4.10. Statistical Analysis

For the behavioral test, results are given as mean ± standard deviation (SD). One-way and two-way analysis of variance, followed by the Tukey and Sidak post hoc test, respectively, were used for the statistical analysis. Sometimes the *t*-test was also used. Results for the in vitro experiments are given as the mean ± SD of three independent triplicates. One-way ANOVA, followed by the Tukey post hoc test, was used for determining the differences between each experimental group and a *p*-value lower than 0.05 was considered significant. All statistical analyses were performed using GraphPad Prism version 5.0 (GraphPad Software Inc., San Diego, CA, USA).

## 5. Conclusions

The present findings show that HSE is a selective anxiolytic agent devoid of antidepressant-like properties at doses able to counteract anxiety-related behavior. In addition, the lack of locomotor impairment, sedative behavior or cognitive impairment indicates a good tolerability profile for HSE. Investigations into the mechanism of action showed the ability of HSE to counteract the decrease in the phosphorylation of ERK44/42, to restore BDNF expression and to return the expression of CREB to basal levels in noradrenergic hippocampal neurons, which indicates it has a protective role against anxiety behavior. These results suggest that oral administration of HSE might represent an interesting opportunity for the management of anxiety disorders.

## Abbreviations

AMI	amitriptyline
BDNF	brain-derived neurotrophic factor
CNS	central nervous system
CREB	cyclic AMP response element binding
DIAZ	diazepam
DBH	dopamine β-Hydroxylase
ERK	extracellular signal-regulated kinase
HSE	*Helichrysum stoechas* Moench methanolic extract
LDB	light dark box
MAO-A	monoamine oxidase A
NORT	novel object recognition test
NSFT	novelty suppressed feeding test
PREG	pregabalin
TST	tail suspension test
PCC	Pearson’s correlation coefficient
M1/M2	Mander’s overlap coefficient 1/2
